# Direct current stimulation modulates gene expression in isolated astrocytes with implications for glia-mediated plasticity

**DOI:** 10.1038/s41598-022-22394-8

**Published:** 2022-10-26

**Authors:** Limary M. Cancel, Dharia Silas, Marom Bikson, John M. Tarbell

**Affiliations:** Department of Biomedical Engineering, The City College of New York, Steinman Hall, Room 404C, 160 Convent Ave, New York, NY 10031 USA

**Keywords:** Biomedical engineering, Cellular neuroscience

## Abstract

While the applications of transcranial direct current stimulation (tDCS) across brain disease and cognition are diverse, they rely on changes in brain function outlasting stimulation. The cellular mechanisms of DCS leading to brain plasticity have been studied, but the role of astrocytes remains unaddressed. We previously predicted that during tDCS current is concentrated across the blood brain-barrier. This will amplify exposure of endothelial cells (ECs) that form blood vessels and of astrocytes that wrap around them. The objective of this study was to investigate the effect of tDCS on the gene expression by astrocytes or ECs. DCS (0.1 or 1 mA, 10 min) was applied to monolayers of mouse brain ECs or human astrocytes. Gene expression of a set of neuroactive genes were measured using RT-qPCR. Expression was assessed immediately or 1 h after DCS. Because we previously showed that DCS can produce electroosmotic flow and fluid shear stress known to influence EC and astrocyte function, we compared three interventions: pressure-driven flow across the monolayer alone, pressure-driven flow plus DCS, and DCS alone with flow blocked. We show that DCS can directly modulate gene expression in astrocytes (notably FOS and BDNF), independent of but synergistic with pressure-driven flow gene expression. In ECs, pressure-driven flow activates genes expression with no evidence of further contribution from DCS. In ECs, DCS alone produced mixed effects including an upregulation of FGF9 and downregulation of NTF3. We propose a new adjunct mechanism for tDCS based on glial meditated plasticity.

## Introduction

The investigation of transcranial direct current stimulation (tDCS) as a noninvasive brain stimulation tool spans decades, including both healthy subjects and patients with a range of neurological conditions^1–4^. tDCS induces changes in neuronal activity and promotes changes in long-term plasticity^5–8^. tDCS has been shown to alter the neuronal gene expression of several plasticity-associated genes, notably BDNF^9,10^. BDNF responsiveness impacts sensitivity to tDCS in both human and animal models^5,11^.

Astrocytes, the most numerous glial cell type in the human nervous system, are integral to long term synaptic plasticity, as well as directly undergoing phenotypic plasticity^12^. BDNF is expressed in neuronal cells but also in other cells—notably astrocytes^13^. In vivo studies show tDCS induces large-amplitude astrocytic Ca^2+^ surges^14^, leading to the hypothesis that tDCS-induced astrocytic activity affects the metaplasticity of the cortex. Here we measured the effects of DCS on neuro-active gene expression, including BDNF, on astrocytes in vitro to characterize a glial-mediated plasticity pathway.

The blood–brain barrier (BBB) is formed by a single layer of endothelial cells (EC) around which pericytes and astrocyte foot processes are wrapped. As part of a broader neurovascular-modulation hypothesis^15^, we previously demonstrated that DCS increases permeability of an in vitro BBB model via the mechanism of electroosmosis^16,17^, and increases BBB permeability in the rat brain^18^. In a current flow modeling study, we proposed that current funneling into capillaries results in a > 400 × amplification in the electric field across the BBB^19^. Here we note this current density concentration may also impact astrocytes wrapped around capillaries.

The aim of the present study was to determine the effect of DCS on the gene expression of isolated astrocytes or ECs in vitro. Expression of neuro-active genes was measured immediately and 1-h after application of DCS at 0.1 mA and 1 mA for 10 min. Since DCS-induced electroosmosis imposes fluid shear stress that can in turn alter gene expression^20–23^, we also sought to separate the effects of DCS dependent-on and independent-of fluid shear stress. Our results point to an interplay between gene activation by pressure-driven flow in the absence of DCS, pressure-driven flow combined with DCS (producing electroosmosis), and DCS on its own with flow (and so electroosmosis) blocked. Expanding previous studies on gene induction by tDCS with mixed cell types^9,24–26^, we found that DCS induced a significant upregulation of FOS and BDNF in isolated astrocytes that occurred along-side but did not require electroosmosis. DCS effects on ECs were largely hard to distinguish from those depending on flow induction.

## Materials and methods

### Cell culture

All reagents were from Sigma (St. Louis, MO) unless otherwise indicated. A mouse brain endothelial cell line, bEnd.3, was obtained from American Type Culture Collection (Manassas, VA) and cultured in Dulbecco’s modified Eagle’s medium (DMEM) supplemented with 10% fetal bovine serum (FBS; Hyclone, Logan, UT), 3 mM L-glutamine, and 1% penicillin–streptomycin. bEnd.3s were plated at 6 × 10^4^ cells/cm^2^ in Transwell PET membrane filters (1.1 or 4.67 cm^2^ membrane area, 0.4 µm pores; Corning, Lowell, MA) coated with fibronectin and cultured for 4–5 days before DCS experiments. Experimental media consisted of phenol-red free DMEM supplemented with 1% bovine serum albumin.

Human astrocytes (HA) were obtained from Cell Applications (San Diego, CA) and cultured according to the manufacturer’s instructions. HA were plated at 3 × 10^4^ cells/cm^2^ in Transwell filters coated with fibronectin and cultured for 5–6 days before DCS experiments. Experimental media consisted of HA basal medium (Cell Applications, San Diego, CA) supplemented with 1% bovine serum albumin.

### Direct current stimulation

**Figure 1:**
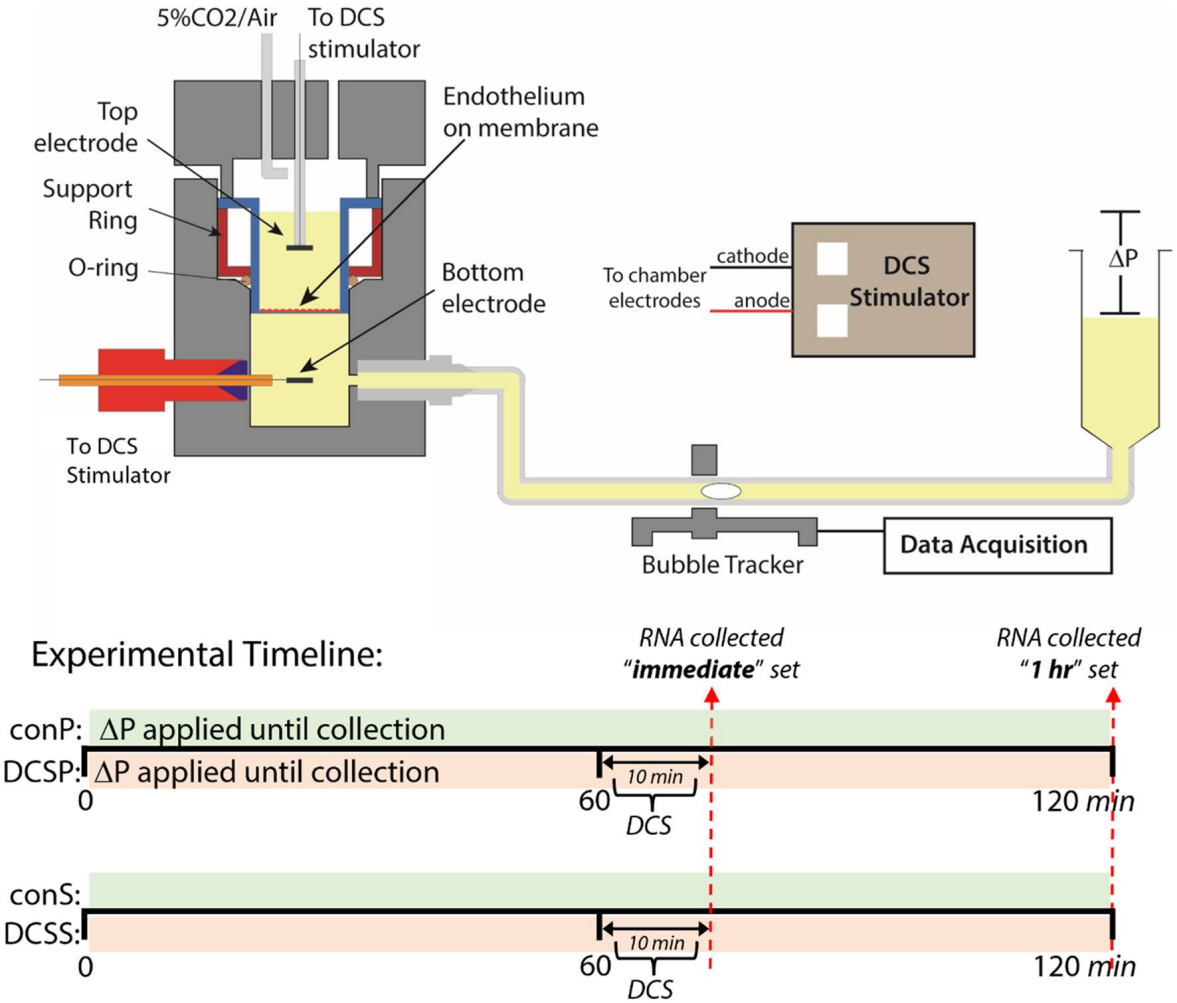
Top: Transport system used to apply direct current stimulation (DCS) to cell monolayers. Transwell filters containing the cell monolayer were sealed inside the chamber. Water flux was measured, via the automated bubble tracker, after the application of a 10 cm H_2_O hydrostatic pressure differential (ΔP). Cell monolayers were stimulated with 0.1–1 mA current for 10 min using a transcranial Direct Current Stimulator. Bottom: Timeline of ΔP and DCS application, and RNA collection, for each experimental group. Note that in the “immediate” collection set of samples, conP and DCSP groups are exposed to ~ 1 h of ΔP, whereas in the “1 h” collection set, conP and DCSP groups are exposed to ~ 2 h if ΔP. Top figure was modified from Sci. Rep. 8, 1–13 (2018).

The Transwell filter containing a bEnd.3 or HA monolayer was inserted and sealed in a custom made transport chamber (Fig. 1), as previously described^16^. Briefly, the transport chamber consisted of luminal and abluminal compartments separated by the cell monolayer. The abluminal compartment was connected to a reservoir that could be lowered to apply a hydrostatic pressure differential that induced convective flow across the monolayer. Water was transported solely across the cell monolayer.

Direct current stimulation was accomplished with a pair of Ag/AgCl electrodes (4 mm x 1 mm disk; A-M Systems, Sequim, WA) positioned 6 mm above and below the cell monolayer. In experiments using the large Transwell membrane, Ag/AgCl electrodes were 8 mm x 1 mm disks positioned 14 mm above and below the cell monolayer. A transcranial Direct Current Stimulator (model 1300-A, Soterix Medical, New York, NY) was used to apply 0.1–1 mA current across the monolayers for 10 min. The current was ramped up to the final value and ramped down to zero over 30 s.

In a typical experiment, the Transwell filter was rinsed twice with experimental medium before being inserted into the transport chamber. DCS was applied in conjunction with a pressure differential that induced transmural fluid flow (shear stress) across the monolayer (DCS P), or static (DCS S) conditions. A static control kept in an incubator for the duration of the experiment was used as the calibrator sample for gene expression analysis. To account for the effect of shear stress on gene expression, a transmural flow control (con P) not exposed to DCS was also included. Each pressure experiment began by lowering the abluminal reservoir to apply a 10 cm H_2_O pressure differential. To ensure that monolayers were intact and sealed, transmural flow (water flux) was measured for 60 min before DCS was applied for 10 min. The con P and DCS P conditions applied a 10 cm H_2_O pressure differential that induced a transmural flow (shear stress) of similar magnitude to that of the electroosmotic flow induced by DCS^16^. The DCS S condition fixed the volume of the system to prevent electroosmotic flow. These conditions made it possible to isolate electroosmotic flow influences from direct current influences. Note that when RNA was collected immediately, pressurized samples (con P and DCS P) were exposed to pressure-driven flow for a total of ~ 1 h, whereas when RNA is collected 1 h after DCS, these samples are exposed to pressure-driven flow for a total of ~ 2 h (see Fig. 1 for experimental timeline).

A note of clarification: In this manuscript we are referring to “shear stress” as that associated with electroosmotic flow induced by DCS. This is flow perpendicular to the monolayer surface induced by fluid that is dragged across the monolayer by the ions carrying the current. This shear stress should not be confused with the shear stress of flowing blood parallel to the surface of endothelial cells*.*

### Real time RT-qPCR

Gene expression was evaluated after a 10-min exposure to DCS with RNA collection performed either immediately after DCS, or 1 h after DCS. Total RNA was extracted using the RNeasy Mini kit (Qiagen, Hilden, Germany) and reversed transcribed to cDNA using the High-Capacity cDNA Reverse Transcription kit (Thermo Scientific, Waltham, MA) according to the manufacturer’s instructions. Real time RT-qPCR was performed on the 7300 Real Time PCR System from Applied Biosystems (Foster City, CA). Tables S1 and S2 list the primers used for bEnd.3 and HA, respectively.

### Protein expression

Western Blot for c-FOS**:** Cells were lysed in RIPA buffer containing 0.1 mL Halt protease inhibitor cocktail (Thermo Scientific) and 0.1 mL phosphatase inhibitor cocktail I (Roche, Basel, Switzerland), scraped, and sonicated. Cell extracts were separated by 12% SDS-PAGE, and proteins were transferred to PVDF membranes. Membranes were blocked with 5% nonfat dry milk for 1 h at room temperature and incubated with primary antibody against c-FOS (1:100, Santa Cruz Biotechnologies; Dallas, TX) overnight at 4 °C. Membranes were incubated with HRP-conjugated anti-mouse IgG secondary antibody for 1 h at room temperature and visualized using the ECL kit (Thermo Scientific). Bands were detected with the ChemiDoc XRS system (BioRad; Hercules, CA). Blots were stripped with Restore stripping buffer (Thermo Scientific), incubated with primary antibody against b-Actin (1:5000), and followed by secondary antibody incubation, visualization and band detection as above. Bands were quantified by densitometry using ImageJ.

### Data analysis

**Figure 2:**
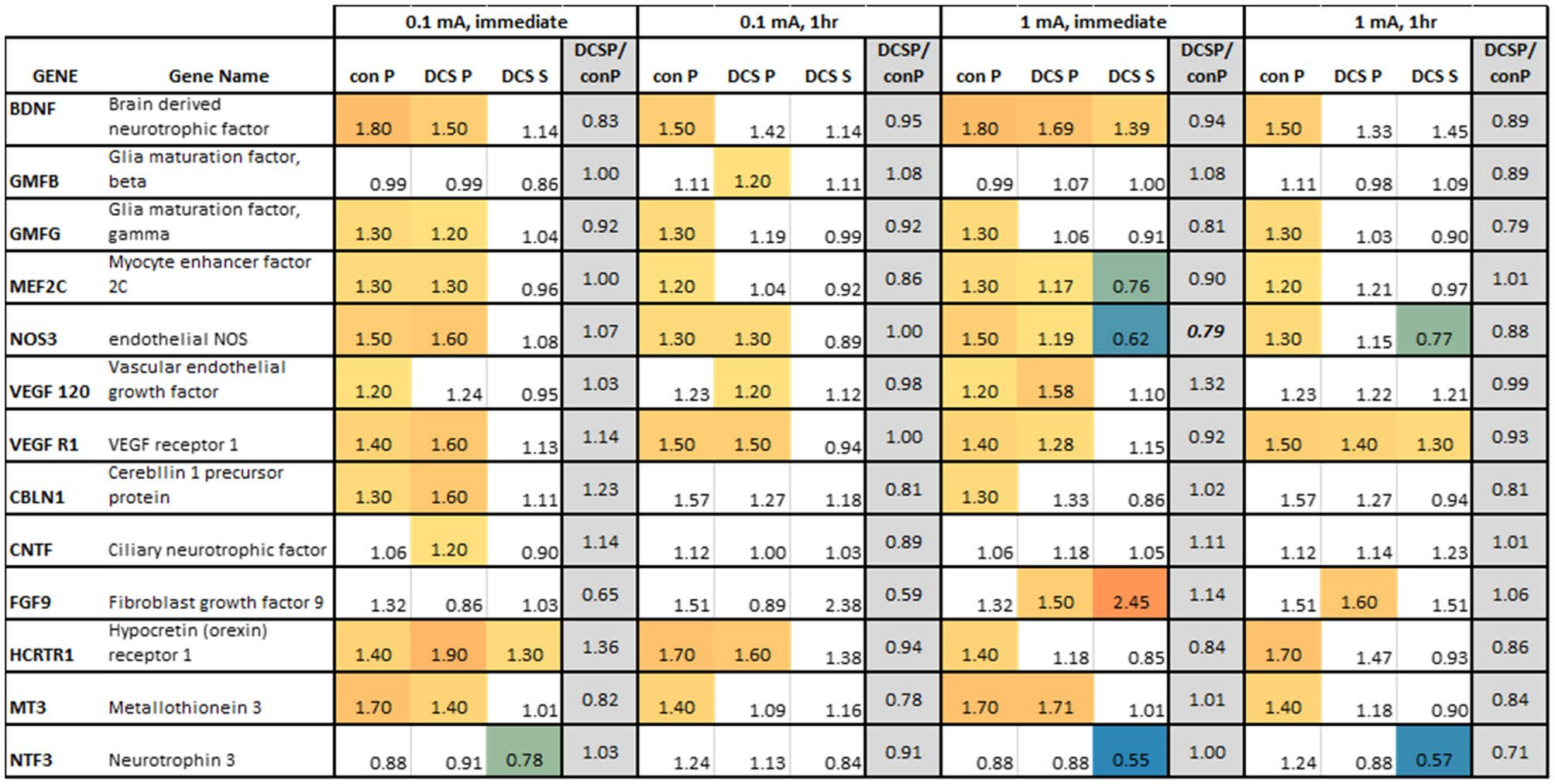
Gene expression for bEnd.3 cells exposed to DCS for 10 min at 0.1 mA or 1 mA with RNA collected immediately or after 1 h. Con P- samples with a hydrostatic pressure gradient that induces convective flow; DCS P- samples with a hydrostatic pressure gradient plus DCS; DCS S-samples under static (no flow) conditions with DCS. Expression shown relative to static control used as calibrator for RT-qPCR. The statistically significant changes are color coded using a 3-color gradient scale where blue to green = downregulation, yellow = onefold, and orange = upregulation, and red =  > fivefold upregulation. N > 7 for all cases. Statistical significance determined by Wilcoxon signed rank test with *p* < 0.05 considered significant. DCS P/con P- shows the fold difference between these samples. Values shown in bold italics denote statistical significance DCS P versus con P by Student’s *t *test with *p* < 0.05 considered significant. Full mean + SEM data set shown in supplementary information Fig. S1.

Relative gene expression data are presented as mean values (Figs. 1 and 2). The mean + SEM data is presented in the supplementary information. GraphPad Prism was used for statistical analyses. For RT-qPCR, con S samples were used as the calibrator and comparisons were made between each group and the hypothetical value of the calibrator (1.0) using a Wilcoxon signed-rank test with *p* < 0.05 considered significant. For protein expression, unpaired two-tailed student’s t-test were used with *p* < 0.05 considered significant.

## Results

### DCS modulation of bEnd.3 gene expression

Thirteen genes were tested in bEnd.3 cells. These are a set of neuroactive genes that are expressed in endothelial cells, as well as some genes known to alter the BBB, such as endothelial nitric oxide synthase (NOS3) and vascular endothelial growth factor (VEGF). Figure 2 shows the relative gene expression after exposure to DCS at 0.1 or 1 mA for 10 min. Statistically significant results are color-coded. Pressure-driven flow alone (con P) induced moderate but significant upregulation in 9 out of the 13 genes, including BDNF which regulates synaptic plasticity and promotes survival of nerve cells. NOS3 and VEGFR1, which can modulate permeability of the BBB^27,28^ also showed significant upregulation. Adding DCS (DCS P) did not induce significant gene expression changes compared to pressure alone, except for NOS3 at 1 mA where a significant downregulation was observed when RNA was collected immediately (see DCS P/con P column in Fig. 2). The largest effect observed was a 2.45-fold upregulation of FGF9, a growth factor for glial cells, by DCS alone (DCS S) after immediate collection. This upregulation was not statistically detectable when RNA was collected 1 h after DCS, nor was any FGF9 upregulation statistically detectable at 0.1 mA. In addition, DCS S at 1 mA induced immediate downregulations of NOS3 (0.62-fold) and NTF3 (0.55-fold), a nerve growth factor, which were sustained 1 h after stimulation. At 0.1 mA, a downregulation of NTF3 (0.78-fold) was observed after immediate RNA collection, but not statistically detectable when RNA was collected 1-h later.

### DCS modulation of astrocyte gene and protein expression

**Figure 3:**
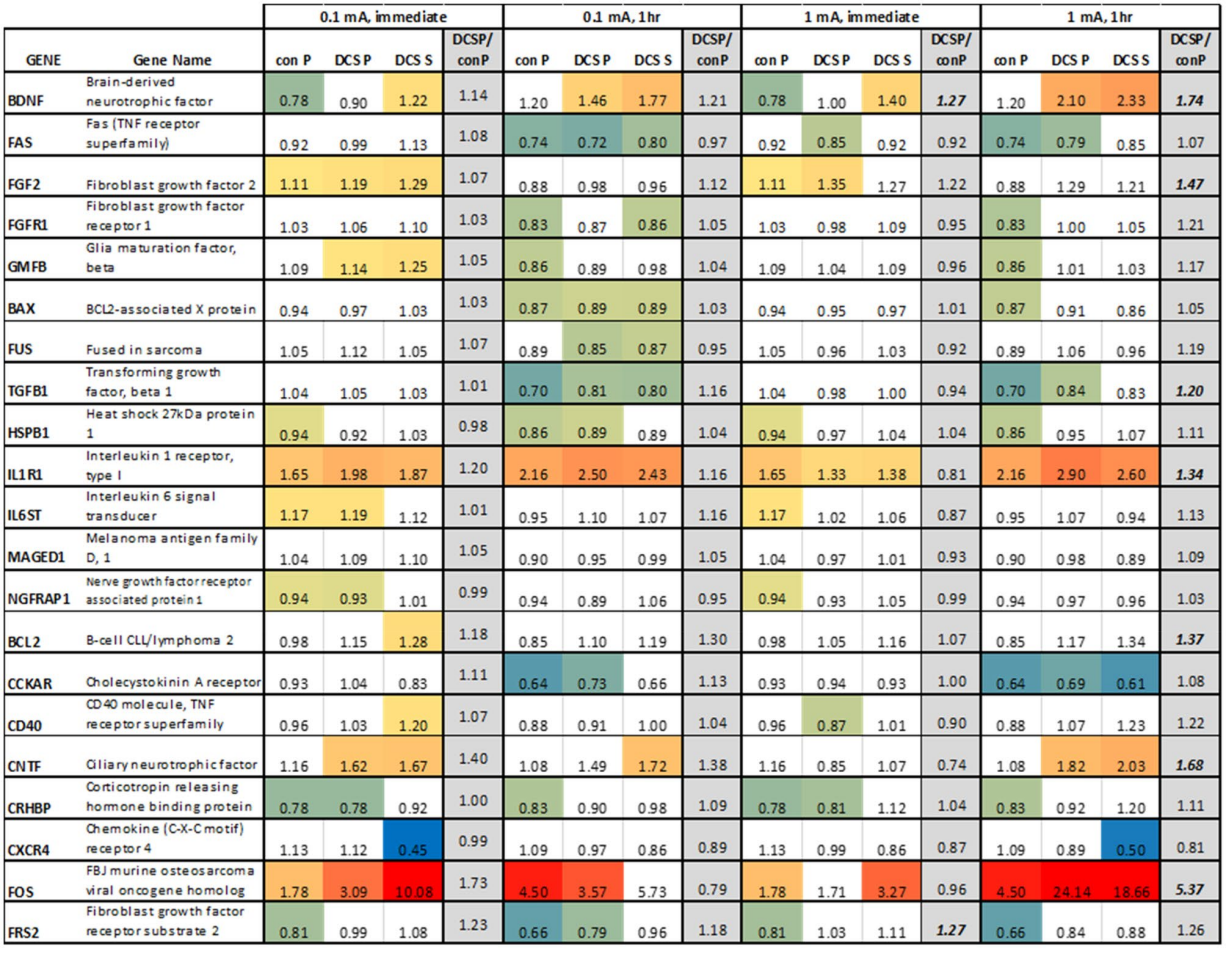
Relative gene expression for human astrocytes exposed to DCS for 10 min at 0.1 mA or 1 mA with RNA collected immediately or after 1 h. Con P- samples with a hydrostatic pressure gradient that induces convective flow; DCS P- samples with a hydrostatic pressure gradient plus DCS; DCS S- samples under static conditions with DCS. Expression shown relative to static control used as calibrator for RT-qPCR. The statistically significant changes are color coded using a 3-color gradient scale where blue to green = downregulation, yellow = onefold, orange = upregulation, and red =  > fivefold upregulation. N > 7 for all cases. Statistical significance determined by Wilcoxon signed rank test with p < 0.05 considered significant. DCS P/con P – shows the fold difference between these samples. Values shown in bold italics denote statistical significance DCS P versus con P by Student’s *t *Test with p < 0.05 considered significant. Full mean + SEM data set shown in supplementary information Fig. S2.

Twenty-one neuroactive genes were tested on human astrocytes. Figure 3 shows the relative gene expression after exposure to 0.1 or 1 mA DCS for 10 min. Statistically significant results are color-coded. Pressure-driven flow (con P) modulated the expression of 15 out of the 21 genes tested. While most of the changes were very small, we note upregulation of 1.65 and 1.78-fold for IL1R1 and FOS, respectively, after immediate RNA collection (1 h of pressure-driven flow in total). These upregulations further increase to 2.16 and 4.50-fold when RNA was collected one hour later (2 h of pressure-driven flow in total). Adding DCS to pressure-driven flow did not induce marked changes compared to pressure alone under most conditions tested. At 1 mA with RNA collected 1 h later, however, several genes showed significant upregulation when DCS was added to pressure-driven flow (see DCS P/con P column in Fig. 3), most notably BDNF (1.74-fold increase) and FOS (5.37-fold increase). There were large upregulations in FOS when DCS was applied with or without pressure-driven flow. At 0.1 mA, FOS was upregulated 3.09-fold under DCS P conditions, and 10.08-fold under DCS alone when RNA was collected immediately. At 1 mA, FOS was upregulated 24.14-fold under DCS P conditions, and 18.66-fold under DCS alone when RNA was collected one hour after stimulation. Interestingly, with immediate RNA collection, BDNF was slightly downregulated by pressure-driven flow alone and upregulated by DCS alone at both current magnitudes. When RNA was collected 1 h later, there was a small, not statistically significant, upregulation by pressure-driven flow and a statistically significant upregulation by DCS alone. For 1 mA, DCS plus pressure-driven flow produced a statistically significant upregulation in BDNF compared to pressure-driven flow alone at both RNA collection times.

**Figure 4:**
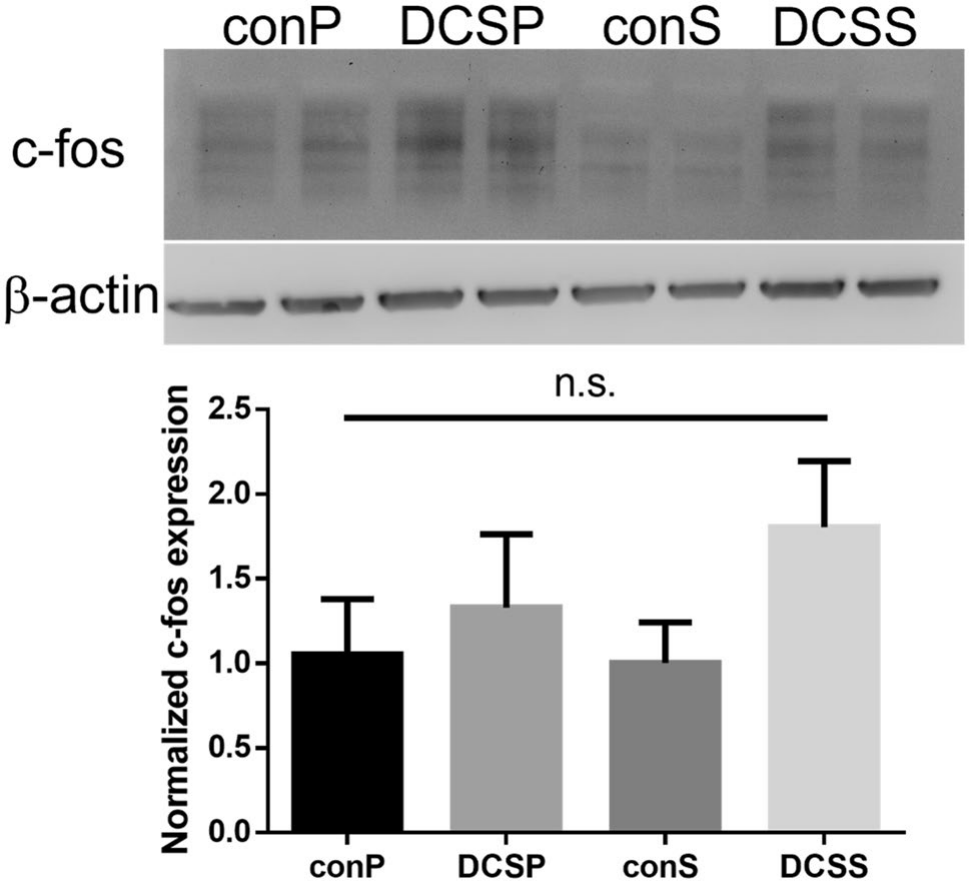
Normalized c-Fos protein expression in human astrocytes and representative western blot. Contrast and brightness on blot image have been altered for visibility, and the blots have been cropped. Unaltered, full-length blots presented in supplemental Fig. S3. n.s = not significant; N = 3 for each group.

Because of the large upregulation in FOS gene expression, we investigated whether c-FOS protein expression was also modulated by DCS using western blot at 1 mA with protein collection performed 1 h after stimulation. Figure 4 shows representative images of the blots and quantification by densitometry. Although there was a trend towards upregulation of c-FOS protein expression by DCS, it did not reach statistical significance. One limitation is that the baseline expression of c-FOS is very low, necessitating large amounts of concentrated protein to get a signal, which we were not always able to obtain from each experiment.

## Discussion

The goal of this study was to investigate whether DCS can alter gene expression in isolated astrocytes and ECs that form the BBB. We exposed ECs and astrocytes to DCS at 1 mA or 0.1 mA for 10 min and quantified changes in gene expression using RT-qPCR. The effects of tDCS on cellular targets range from acute (within minutes) to long-term^6,14^. Given the limitations of maintaining a cell culture long term in our experimental setup, we investigated the acute effects of DCS on gene expression. RNA was collected immediately after a 10-min DCS exposure, with the aim of capturing acute changes induced by the electric field itself and/or electroosmosis. To determine whether these changes were dependent on the presence of the electric field (as is electroosmosis) or more lasting in nature, total RNA was also collected one hour after stimulation.

The following conditions were included in this study: a static control condition without pressure-driven flow or DCS (conS), which served as the calibrator for gene expression analysis, a pressure-driven flow without DCS control condition (conP), a DCS with flow blocked condition (DCS S), and the physiological condition of pressure (flow) plus DCS (DCS P). The conS condition is not a physiological one. Recent studies support the idea that bulk flow is an important mechanism of transport in the brain parenchyma^29^. Therefore, both endothelial cells and astrocytes are likely to experience continuous fluid flow (shear stress) in vivo. More specifically, in this study we tried to simulate the interstitial flow on astrocytes associated with electroosmotic flow induced by DCS. However, including the static condition along with the conP condition allowed us to characterize whether the observed changes by DCS were due to fluid flow alone. We previously showed DCS induces fluid flow via electroosmosis and that the amount of induced flow is about the same as that induced by a 10 cm H_2_O pressure differential^16^. It is established that increased fluid shear stress around the cells can alter gene expression^20–23^. Including static and pressurized samples with and without DCS allowed us to evaluate whether flow and DCS acted synergistically, or if their effects on each gene were in opposition.

In ECs, we report that pressure-driven flow without DCS induced statistically significant changes in the expression of 9 out of 13 genes tested in bEnd.3 cells. None of the changes were above twofold and adding DCS with pressure-driven flow did not induce notable further changes. In ECs, DCS alone (with flow blocked) induced an upregulation of BDNF and a 2.45-fold increase in FGF9 at 1 mA when RNA was collected immediately. DCS alone downregulated ECs expression of NTF3, NOS3, MEF2C; NTF3 and NOS3 persisting for 1 h at 1 mA. MEF2C and NOS3 changes were qualitatively in a different direction than flow driven changes (con P). The role of endothelial NTF3 beyond fetal development has not been fully investigated. However, it’s been shown that NTF3 induces nitric oxide (NO) in rat cerebral endothelial cells^30^, and in subependymal neural stem cells (NSCs)^31^. It was also shown that NTF3-induced NO acts as a cytostatic factor, promoting quiescence and long-term maintenance of NSCs^31^. Taken together these results point to nuanced effects of DCS on EC gene expression that can both derive from or occur independent of induced flow.

For astrocytes, we report pressure-driven flow without DCS (con P) induced significant gene expression changes in 15 out of 21 genes, with the highest relative increases in FOS and IL1R1 and several genes downregulated. Adding DCS with pressure-driven flow did not result in marked further changes, except for FOS and BDNF. DCS alone (with flow blocked) impacted several genes notably an increasing expression of FOS, IL1R1, CNTF, and BNDF. As with ECs, effects of DCS on gene expression in astrocytes points to sophisticated effects involving both flow-dependent and flow-independent changes. For example, astrocyte BDNF expression is enhanced by both flow-independent mechanisms and by flow-dependent (electroosmotic) mechanisms.

Previous studies with mixed cell types have shown that tDCS induces expression changes of several immediate early genes in neurons including FOS^9,24–26^. Immediate early genes are activated rapidly upon cell stimulation, before any protein synthesis has occurred, and serve as regulators of downstream target genes^32^. The dimeric complex of FOS and JUN proteins forms the transcription factor activator protein-1, AP-1, which binds to DNA and has a role in coupling extracellular stimuli with changes in gene expression^33^. In neuronal tissue, FOS upregulation is routinely used as a marker of activated neurons^25,32,34^ and has been associated with learning and memory^32,35,36^. In cultured astrocytes, FOS expression is also a marker of activation^37^ and has been associated with differentiation and proliferation^38^.

To our knowledge, the present study is the first to show gene upregulation in astrocytes, including FOS and BDNF, in response to DCS. However, prior studies using long-term electrical stimulation have shown activation of astrocytes in vitro. Neurons and astrocytes cultured on microelectrode arrays migrated toward the stimulating electrode after 24 h of stimulation^39^. It was observed that migration was induced earlier in neuron and astrocyte cocultures than in neuron-only cultures, and the viability of neurons was enhanced in cocultures^39,40^. Using FOS as a marker of activation, continuous motor cortex stimulation in rats was shown to induce the number of astrocytes in the cortex^41^. Previous studies have shown that astrocytes are necessary for long-term potentiation and plasticity^42–44^. A recent study in mice showed that astrocyte activation, without neuronal activation, induces long-term potentiation and enhances memory allocation^45^. Using a mouse model of tDCS, Monai et al. observed elevation of astrocytic but not neuronal Ca^2^^+^, leading to synaptic plasticity and improvement of depression-like behavior^14^.

This study had several limitations inherent to the approach adopted. The application of current across the monolayers is not directly analogous to application of current across the brain. In the in vivo BBB, endothelial cells do form a monolayer, and while astrocytes don’t form a monolayer in vivo, the astrocyte foot processes surrounding endothelial cells resemble a monolayer (see for example: Fig. 2A in Kutusov et al.^46^). We also note that the interaction between endothelial cells and astrocytes is not captured in our model (no co-culture). But a previous study of co-culture models of endothelial cells and astrocytes^47^ showed little interaction effect on the permeability of a BBB model and displayed trends that closely followed in vivo measurements in the rat pial circulation. It would, however, be valuable to look at gene expression changes in cells isolated from mice exposed to DCS. It would also be important to extend the scope of this study by using broad RNA-Seq technology.

With the goal of clearly isolating action on cell types, we do not access secondary inter-cell-type interactions (e.g., how astrocyte BDNF would impact neuronal plasticity). Extending these experiments to in vivo models would be difficult to interpret precisely because of their functional coupling^15,48–50^. There is a large space of stimulation waveform to explore (e.g., duration), and we start here with intensity. We cannot explain the non-linearity in cellular dose response; however, non-linear dose response is observed in tDCS^51–55^ and our results show this can originate even at the astrocyte or endothelial cellular level. Indeed, complex dose response of astrocytes and endothelial cells to chemical and mechanical signals is common.

In conclusion, this study shows that DCS modulates gene expression in both endothelial cells and astrocytes. In ECs more of the tested genes were responsive to convective flow, whether only pressure driven or with DCS, including BDNF, NOS3, and VEGFR1. In astrocytes, there was more evidence for interactions between flow-dependent and flow-independent changes in tested gene expression, including of FOS and BDNF. The largest effects observed were for the immediate early gene FOS (as much as 24-fold increase). We have previously shown that DCS induces increased flow across EC monolayers due to the electroosmotic effect^16^ but direct effects of electric field on ECs and astrocyte polarization are plausible. While the cellular and molecular targets of tDCS still continue to be explicated, our results support the idea that endothelial cells and astrocytes forming the BBB are probable targets, and their responses may, in part, explain the changes (including plasticity) of neuronal activity produced by tDCS.

## Supplementary Information

Supplementary Information 1.

Supplementary Information 2.

Supplementary Information 3.

## Data Availability

The datasets generated and/or analyzed during the current study are included in this article, its supplemental files, and available in the Gene Expression Omnibus repository (GSE207140, https://www.ncbi.nlm.nih.gov/geo/query/acc.cgi?acc=GSE207140).
