# Supplementary material for: Direct current stimulation modulates gene expression in isolated astrocytes with implications for glia-mediated plasticity
Source: Sci Rep. 2022 Oct 26;12:17964. doi: 10.1038/s41598-022-22394-8 (PMC9606293; doi:10.1038/s41598-022-22394-8)

**Supplementary Information**

**Table S1. Primers used for bEnd.3 gene expression.**

| Symbol | Gene Name | Forward | Reverse |
| --- | --- | --- | --- |
| Bdnf | Brain derived neurotrophic factor | TCATACTTCGGTTGCATGAAGG | AGACCTCTCGAACCTGCCC |
| Gmfb | Glia maturation factor, beta | AGAAACCCACAATGCTGCTATT | TACGAGACTCTGCCATCATCA |
| Gmfg | Glia maturation factor, gamma | AATGCCGCCATCATAATGAAAGT | GCTGTCTCTCTGGCAATTCCA |
| Mef2c | Myocyte enhancer factor 2C | ATCCCGATGCAGACGATTCAG | AACAGCACACAATCTTTGCCT |
| Nos3 | endothelial NOS | CACCAGGAAGAAGACCTTTAAGGA | CACACGCTTCGCCATCAC |
| Vegf 120 | Vascular endothelial growth factor | GCCAGCACATAGAGAGAATGAGC | CGGCTTGTCACATTTTTCTGG |
| Vegfr1 | Vegf receptor | GCCAGACTCTCTTTCTCAAGTGC | GGGGAGTGATGCTCAGCCT |
| Cbln1 | Cerebllin 1 precursor protein | TCTGACCCTACGGGCACTG | TCTCGGACGGCTCATGGTT |
| Cntf | Ciliary neurotrophic factor | TCTGTAGCCGCTCTATCTGG | GGTACACCATCCACTGAGTCAA |
| Fgf9 | Fibroblast growth factor 9 | ATGGCTCCCTTAGGTGAAGTT | TCCGCCTGAGAATCCCCTTT |
| Hcrtr 1 | Hypocretin (orexin) receptor 1 | CTGTGGCGCGATTATCTCTAC | GCCAGGGACAGGTTGACAA |
| Mt3 | Metallothionein 3 | ACCTGCCCCTGTCCTACTG | CCTTGGCACACTTCTCACATC |
| Ntf3 | Neurotrophin 3 | GGAGTTTGCCGGAAGACTCTC | GGGTGCTCTGGTAATTTTCCTTA |

**Table S2. Primers used for Human Astrocyte gene expression.**

| Symbol | Gene Name | Forward | Reverse |
| --- | --- | --- | --- |
| BDNF | Brain-derived neurotrophic factor | CTACGAGACCAAGTGCAATCC | AATCGCCAGCCAATTCTCTTT |
| FAS | Fas (TNF receptor superfamily, member 6) | TCTGGTTCTTACGTCTGTTGC | CTGTGCAGTCCCTAGCTTTCC |
| FGF2 | Fibroblast growth factor 2 (basic) | AGAAGAGCGACCCTCACATCA | CGGTTAGCACACACTCCTTTG |
| FGFR1 | Fibroblast growth factor receptor 1 | GGCTACAAGGTCCGTTATGCC | GATGCTGCCGTACTCATTCTC |
| GMFB | Glia maturation factor, beta | ATGTTGCCGAAGATTTAGTGGAA | CCACCAGGCGTTTATCCTTGT |
| BAX | BCL2-associated X protein | CCCGAGAGGTCTTTTTCCGAG | CCAGCCCATGATGGTTCTGAT |
| FUS | Fused in sarcoma | TCAATCCTCCATGAGTAGTGGT | CACGGTCCTGCTGTCCATA |
| TGFB1 | Transforming growth factor, beta 1 | CAATTCCTGGCGATACCTCAG | GCACAACTCCGGTGACATCAA |
| HSPB1 | Heat shock 27kDa protein 1 | ACGGTCAAGACCAAGGATGG | AGCGTGTATTTCCGCGTGA |
| IL1R1 | Interleukin 1 receptor, type I | GGCCAGTTGAGTGACATTGCT | TGTGATGAGGGTACTCCTTCTTT |
| IL6ST | Interleukin 6 signal transducer (gp130, oncostatin M receptor) | GTGAGTGGGATGGTGGAAGG | CAAACTTGTGTGTTGCCCATTC |
| MAGED1 | Melanoma antigen family D, 1 | GAAGACAGCGCCTTGCTTATG | GTGGCCTGGTTAGTAGGTGG |
| NGFRAP1 | Nerve growth factor receptor (TNFRSF16) associated protein 1 | GCAGCCTATGCAGAATGGAGA | CCCCATAAGGATACGCAGACA |
| BCL2 | B-cell CLL/lymphoma 2 | GGTGGGGTCATGTGTGTGG | CGGTTCAGGTACTCAGTCATCC |
| CCKAR | Cholecystokinin A receptor | CCCATGCTTTGAAGGTGATTGC | CATATTCGCGGTCTGGTTGTTA |
| CD40 | CD40 molecule, TNF receptor superfamily member 5 | ACTGAAACGGAATGCCTTCCT | CCTCACTCGTACAGTGCCA |
| CNTF | Ciliary neurotrophic factor | ACAGAGCATTCACCGCTGAC | TCAGGTCTGAACGAATCTTCCTT |
| CRHBP | Corticotropin releasing hormone binding protein | CACACCAGCATCGAAACTGC | TGAAGACCATTTACGTGTCCCA |
| CXCR4 | Chemokine (C-X-C motif) receptor 4 | ACGCCACCAACAGTCAGAG | AGTCGGGAATAGTCAGCAGGA |
| FOS | FBJ murine osteosarcoma viral oncogene homolog | CCGGGGATAGCCTCTCTTACT | CCAGGTCCGTGCAGAAGTC |
| FRS2 | Fibroblast growth factor receptor substrate 2 | CCTGCGACGCTATGGCTATG | ACGGGCACACTTAAAGGCAAA |


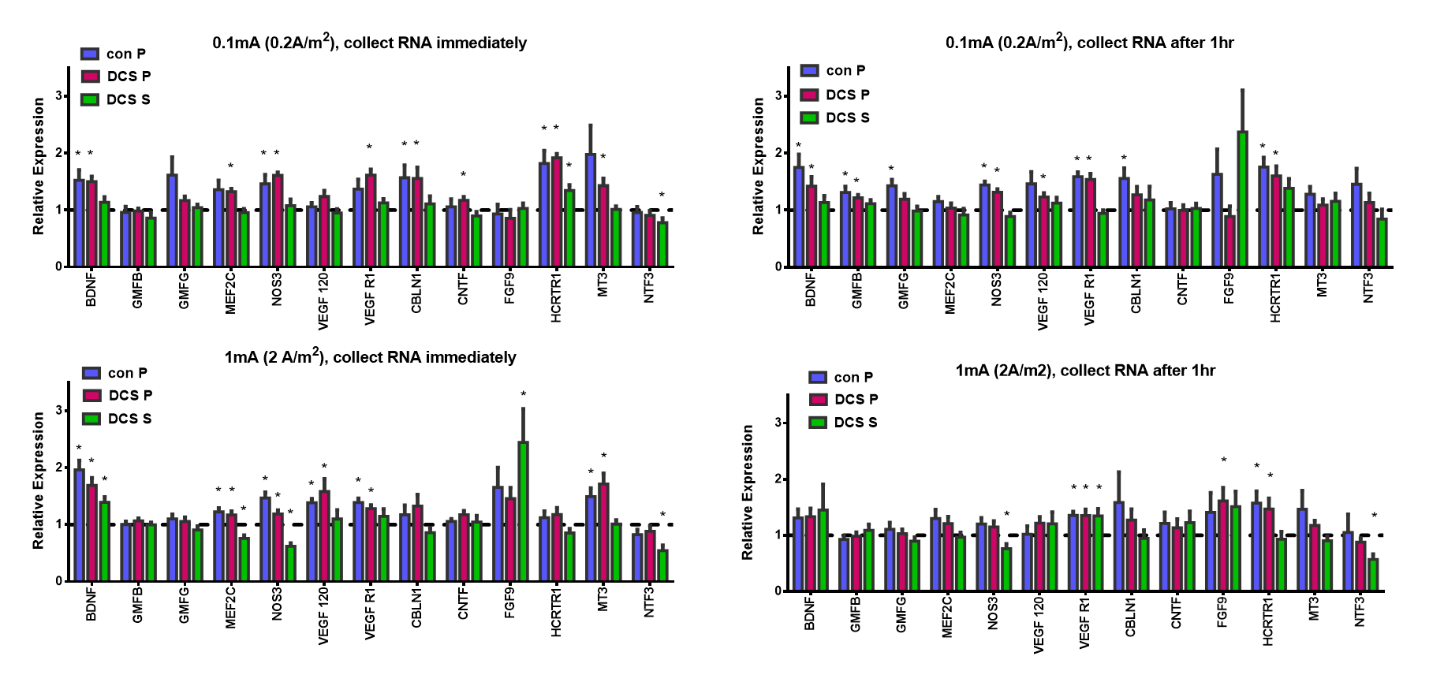


**Figure S1.** Gene expression for bEnd.3 cells exposed to DCS for 10 min at 0.1mA (top) or 1mA (bottom); RNA collected immediately (left) or after 1hr (right). ConP- Samples with a hydrostatic pressure gradient that induces convective flow; DCS P- samples with a hydrostatic pressure gradient plus DCS; DCS S- samples under static conditions with DCS. All samples are normalized to static control (ConS; represented by dashed line at 1.0). n>7 for all cases. *Significantly different from 1.0 by Wilcoxon signed rank test, p<0.05.

**Figure S2.** Gene expression for human astrocytes exposed to DCS for 10 min at 0.1mA (top) or 1mA (bottom); RNA collected immediately (left) or after 1hr (right). ConP- Samples with a hydrostatic pressure gradient that induces convective flow; DCS P- samples with a hydrostatic pressure gradient plus DCS; DCS S- samples under static conditions with DCS. All samples are normalized to static control (ConS; represented by dashed line at 1.0). n>7 for all cases. *Significantly different from 1.0 by Wilcoxon signed rank test, p<0.05.

**Figure S3.** Unaltered, full-length images of western blots, and bright field image of ladder for each blot.


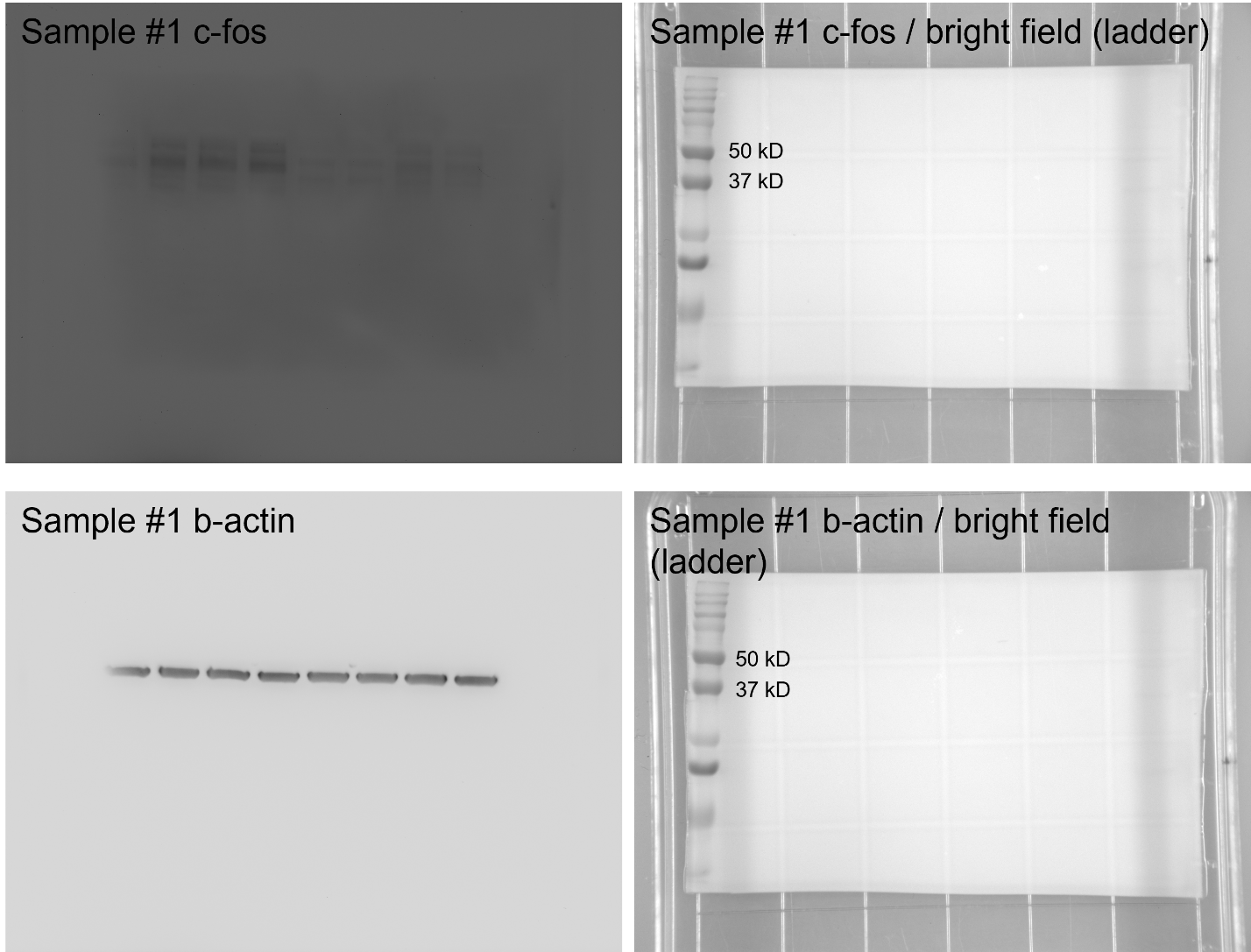


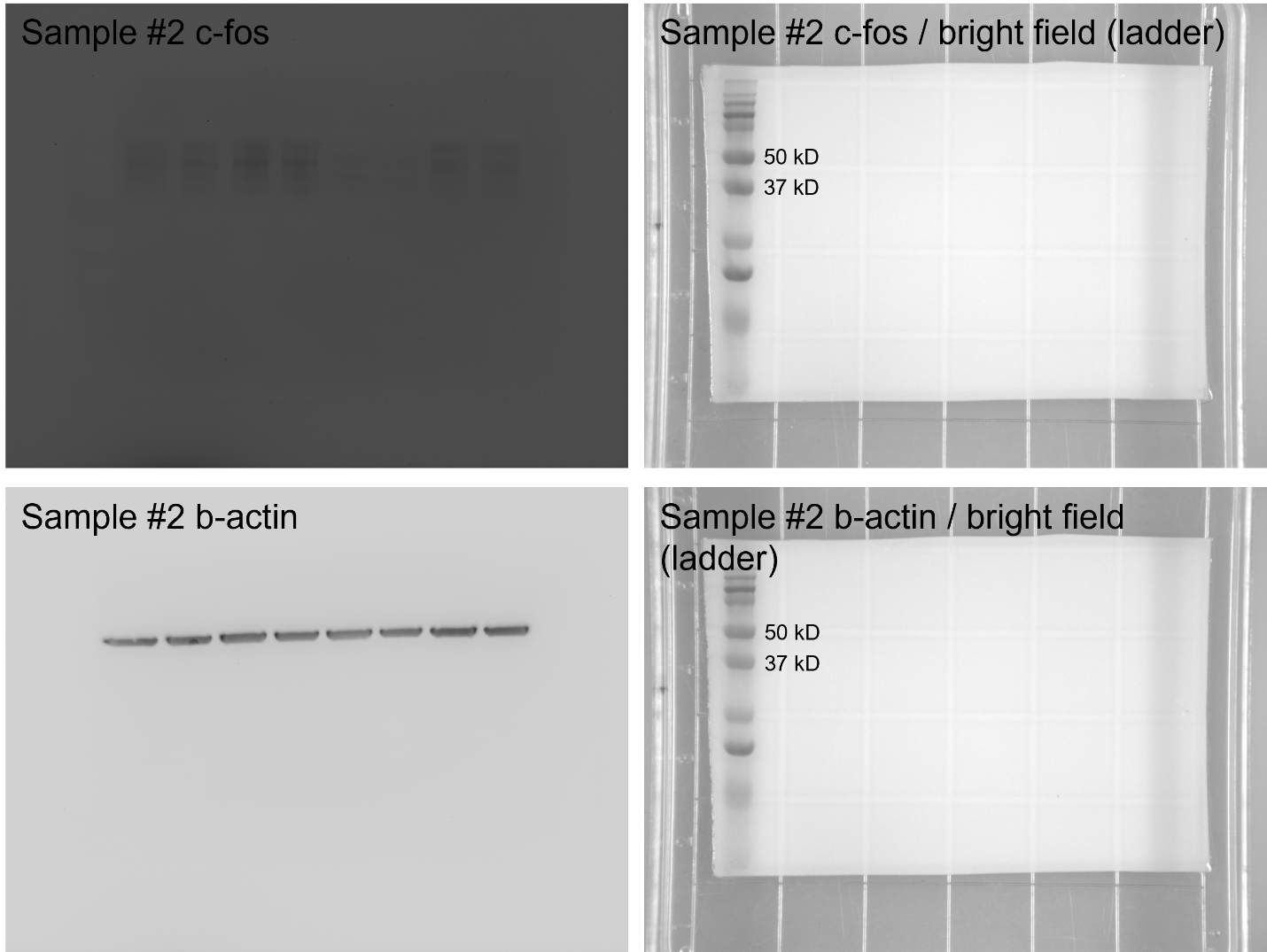


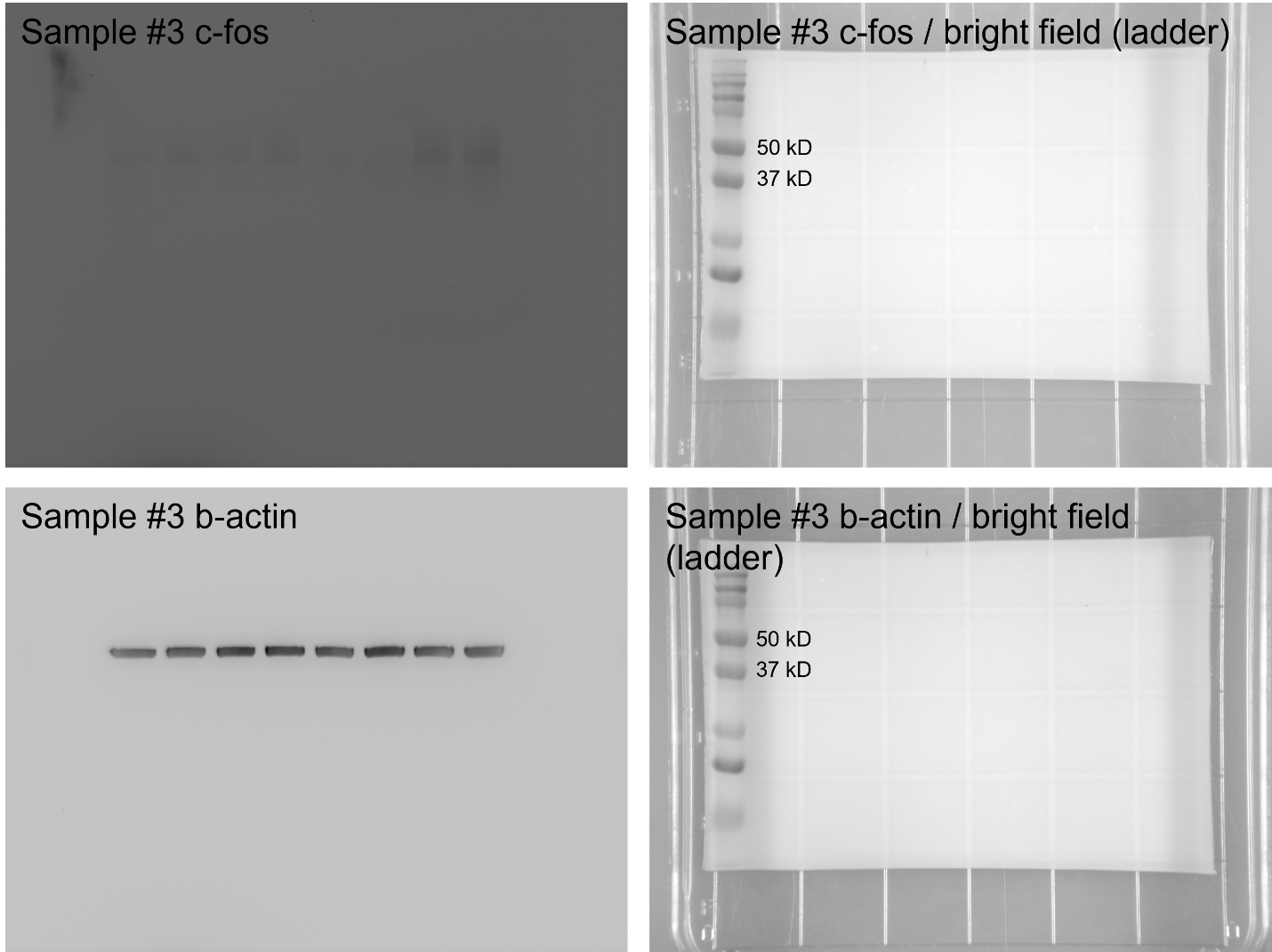

Supplement: Supplementary file 3 — Supplementary Information 3. [file 41598_2022_22394_MOESM3_ESM.docx]
